# Population differentiated copy number variation of *Bos taurus*, *Bos indicus* and their African hybrids

**DOI:** 10.1186/s12864-021-07808-7

**Published:** 2021-07-12

**Authors:** Jisung Jang, Endashaw Terefe, Kwondo Kim, Young Ho Lee, Gurja Belay, Abdulfatai Tijjani, Jian‑Lin Han, Olivier Hanotte, Heebal Kim

**Affiliations:** 1 Interdisciplinary Program in Bioinformatics, Seoul National University, Seoul, Republic of Korea; 2 Addis Ababa University, MCMB Department, Addis Ababa, Ethiopia; 3 International Livestock Research Institute (ILRI), Addis Ababa, Ethiopia; 4 Arsi University, Asella, Ethiopia; 5 Department of Agricultural Biotechnology and Research Institute of Agriculture and Life Sciences, Seoul National University, Seoul, Republic of Korea; 6 The Centre for Tropical Livestock Genetics and Health (CTLGH), The Roslin Institute, The University of Edinburgh, Edinburgh, UK; 7 CAAS-ILRI Joint Laboratory on Livestock and Forage Genetic Resources, Institute of Animal Science, Chinese Academy of Agricultural Sciences (CAAS), Beijing, China; 8 Livestock Genetics Program, International Livestock Research Institute (ILRI), Nairobi, Kenya; 9 School of Life Sciences, University of Notting‑ ham, Nottingham, UK; 10 eGnome, Inc., Seoul, South Korea

**Keywords:** Copy number variation (CNV), Indicine, Taurine, African indigenous cattle, Population differentiated CNV

## Abstract

**Background:**

CNV comprises a large proportion in cattle genome and is associated with various traits. However, there were few population-scale comparison studies on cattle CNV.

**Results:**

Here, autosome-wide CNVs were called by read depth of NGS alignment result and copy number variation regions (CNVRs) defined from 102 Eurasian taurine (EAT) of 14 breeds, 28 Asian indicine (ASI) of 6 breeds, 22 African taurine (AFT) of 2 breeds, and 184 African humped cattle (AFH) of 17 breeds. The copy number of every CNVRs were compared between populations and CNVRs with population differentiated copy numbers were sorted out using the pairwise statistics *V*_*ST*_ and *Kruskal-Wallis* test. Three hundred sixty-two of CNVRs were significantly differentiated in both statistics and 313 genes were located on the population differentiated CNVRs.

**Conclusion:**

For some of these genes, the averages of copy numbers were also different between populations and these may be candidate genes under selection. These include olfactory receptors, pathogen-resistance, parasite-resistance, heat tolerance and productivity related genes. Furthermore, breed- and individual-level comparison was performed using the presence or copy number of the autosomal CNVRs. Our findings were based on identification of CNVs from short Illumina reads of 336 individuals and 39 breeds, which to our knowledge is the largest dataset for this type of analysis and revealed important CNVs that may play a role in cattle adaption to various environments.

**Supplementary Information:**

The online version contains supplementary material available at 10.1186/s12864-021-07808-7.

## Background

Cattle (*Bos taurus*) has been an invaluable animal providing livestock products such as milk, meat, leather and acting as a draft animal for cultivation and transportation since the domestication of extinct wild aurochs (*Bos primigenius*) [1]. The two subspecies of *Bos taurus*, taurine (*B. t. taurus*) and zebu (*B. t. indicus*) were brought about after bifurcation in 335,000 BP, and were domesticated independently in different time and location [2, 3]. Archaeological and genomic evidences indicate that the taurine was domesticated approximately 10,000 YBP in Fertile Crescents and the zebu was domesticated 8000 YBP in Indus Valley [4–6]. The domesticated cattle populations were dispersed quickly after domestication along with the migration of pastoralists [5]. Their adaption to various local environments, artificial selection and introgression gave rise to genetically and phenotypically diversified modern cattle breeds [7].

Genome-wide variations such as SNPs and small INDELs of cattle were identified in previous studies [8, 9]. These small variations have been studied for understanding cattle evolution including population structure, selection, demographic history and introgression [7, 10, 11]. In case of structural variation, a large proportion in the genome is comprised of CNVs which have great effects on changing of gene structure, dosage and expression level [12, 13]. In spite of its potentially high functional effects and abundance in the genome, insufficient data and absence of standards in detection and downstream analysis make understanding of CNVs and their impact in cattle genome difficult. However, recent releases of high quality cattle genome assemblies such as ARS-UCD1.2, UOA_Angus_1 and UOA_Brahman_1 make NGS based CNV study available and more credible [14, 15]. The CNV calling based on short read mapping is now able to detect rare or novel variants, expanded target region to genome-wide, and improved resolution of the location [16].

Here, we detected genome-wide CNVs of 336 individuals in 39 global cattle breeds including Eurasian taurine, Asian indicine and African indigenous cattle, and 2 individuals of African buffalo (*Syncerus caffer caffer*) using NGS read mapping. This is the largest number of breeds and individuals used in an NGS read mapping based cattle CNV study, including, notably, 19 breeds of African cattle that have not been well understood in terms of their CNVs. CNVs were defined from paired-end mapping result of short reads produced by Illumina HiSeq or NovaSeq platform. We performed population genetics survey on autosomal copy number variation regions (CNVRs). Hierarchical clustering of CNVRs from all individuals were compared to geographical origins and breeds. CNVRs with population differentiated copy number were identified by pairwise comparison of variance and rank based statistics. Population differentiated CNVRs overlapping genes were functionally annotated and suggested as candidate genes associated with selection and adaptation.

## Result

### CNV calling and CNVR definition

The coverage and sequencing depth of mapped short reads data are important to reliably call CNVs using read depth information. In several previous studies, samples with mean depth coverage over 5x were used for CNV analysis, showing that 4x depth coverage is sufficient for read depth-based CNV detection [17–19]. In our dataset, the minimum mean depth was higher than 5.1x, and the mean values of alignment rate, coverage and mean depth of coverage were 99.5, 95.0%, 11.4x (Table S1). After calling and filtering CNVs, 18,391 CNVRs were identified on autosomes (Table S2), covering 236.2 Mbp or 9.49% of *B. taurus* autosomes.

### Population differentiation based on CNVR

**Fig. 1:**
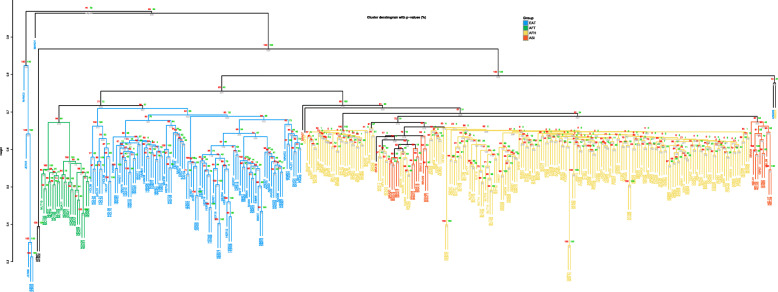
Hierarchical clustering tree. For every individual, the absence or presence of CNVs in autosomal CNVRs was converted to vector made of ‘0’s and ‘1’s. The hierarchical clustering was performed on these vectors representing each individual. The bootstrap value was written under the edges of every clustering. The approximately unbiased (AU) and the bootstrap probability (BP) *p*-value were written in red and green letters on the edges after being multiplied by 100. The branch of hierarchical clustering tree were colored to indicate the group of clades following their region and population such as AFB, AFH, AFT, ASI and EAT

In the hierarchical clustering tree based on CNVR, 8 individuals including 1 Maremmana (MAM03), 1 Maronesa (MAN01), 4 Jersey individuals (JER03, JER04, JER05 and JER06), 1 Angus (ANG09) and 1 Ankole (ANK03) were distant from other individuals (Fig. 1). Except for the 8 individuals, 330 individuals which consisted of 2 AFB, 211 ASI or AFH (indicine group), 117 EAT or AFT (taurine group) were classified by their species and subspecies. Most of the taurine individuals were clustered by their breeds in contrast to indicine individuals. The AFT individuals were clustered by their breeds, and were separated from EAT breeds that were mostly well clustered by their breeds. The four EAT breeds, Holstein, Hanwoo, Hereford and Simmental, were distinguished from other breeds and all individuals in each breed were clustered together. The individuals of two Finn cattle breeds, Western Finn and Eastern Finn, were not distinguished from each other, but clustered together. Six of ten Angus and 9 of 10 Jersey individuals were clustered and differentiated by their breeds. Rest of the taurine individuals included in Maremmana, Podolica, Pajuna, Sayaguesa and Limia from South-Western Europe were clustered together. While Nelore and Gir were distinguished from AFH, individuals in other ASI breeds such as Brahman, Sahiwal, Tharparkar and Hariana were clustered with AFH individuals.

**Fig. 2:**
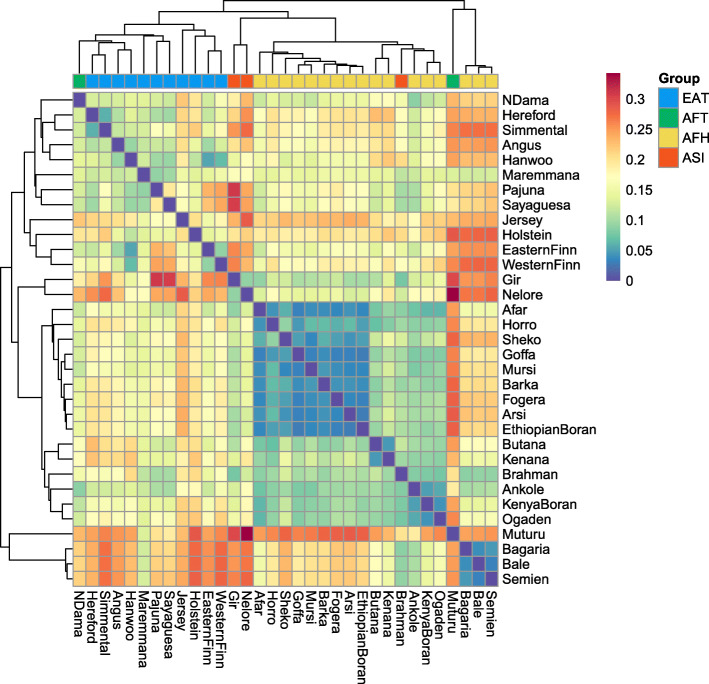
Manhattan plot of *V*_*ST*_. *V*_*ST*_ of CNVRs were visualized as Manhattan plots. The center point of CNVRs was used as x-coordinate value. Differentiated genes overlapped with CNVRs significantly different both in upper 1% *V*_*ST*_ and 0.01 significance level of *Kruskal-Wallis* test on their copy number. The genes whose symbol is starting with ‘*LOC*’ or differentiated in ASI-AFT pair were left out due to lack of space. The upper 1% percentile *V*_*ST*_, 0.500 and upper 0.1% percentile, 0.759 were shown as green and red lines respectively

The variance of copy numbers of each breed and *V*_*ST*_ of every autosomal CNVR were calculated for every breed pairs. The range of *V*_*ST*_ is from 0 to 1, with a higher value indicating a larger difference. The pairwise mean V_ST_ of regional population were as following: EAT-AFT, 0.008; EAT-ASI, 0.017; AFH-ASI, 0.023; AFH-EAT, 0.024; AFH-AFT, 0.045; AFT-ASI, 0.128 (Fig. 2). The average of the mean of pairwise *V*_*ST*_ in breed level was 0.166. Most of the AFH and ASI were clustered together and N’Dama was clustered with EAT. Muturu was clustered with the 3 Ethiopian humped breeds including Bagaria, Bale and Semien, and separated from others. Several groups of breeds originated from adjacent region including Finn taurine (Eastern Finn and Western Finn), and the Ethiopian zebu (Bagaria, Bale and Semien) were clustered together by their mean *V*_*ST*_.

### Detection of candidate CNVR differentiated across populations

**Fig. 3:**
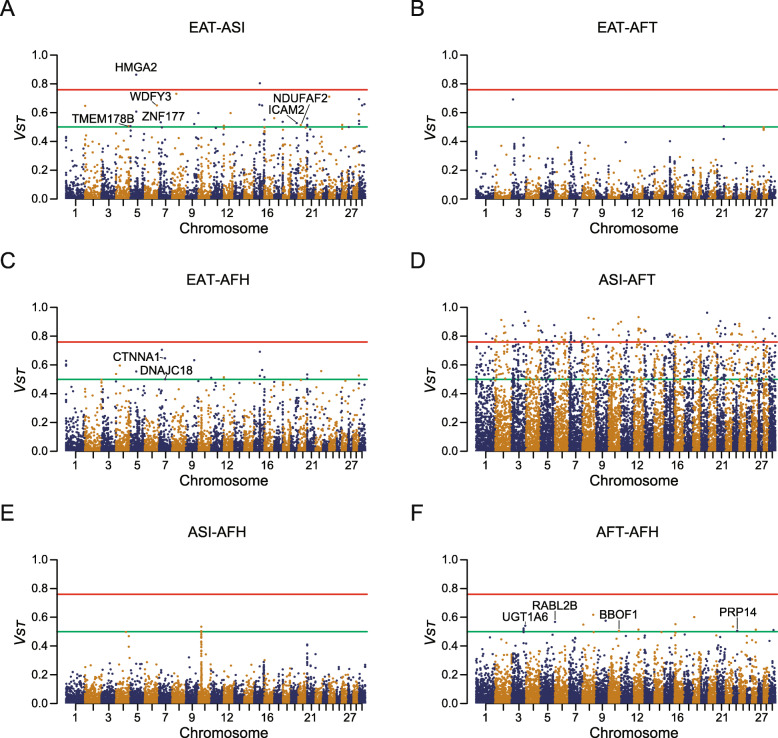
Mean pairwise *V*_*ST*_ values between cattle breeds represented by more than one animal. Clustering tree and heatmap of mean pairwise *V*_*ST*_ of autosomal CNVRs. The group of breed was visualized by color above each column. The arrangement of breeds in row and column followed the order by clustering tree. The agglomeration method of clustering was weighted pair group method with arithmetic mean (WPGMA). Breeds were classified to 4 groups by their originated region and taxonomy as follows; AFH, African Humped cattle; AFT, African humpless taurine; ASI, Asian indicus; EAT, Eurasian taurine

In order to detect population differentiated CNVR across 4 groups (AFH, AFT, ASI, and EAT), two statistics were employed. First, pairwise *V*_*ST*_ were calculated between all populations except for AFB. Top 1% and top 0.1% values were about 0.500 and 0.759, respectively. The number of CNVRs with the top 0.1% *V*_*ST*_ was 109 in ASI-AFT pair, 2 in ASI-EAT pair and 0 in other pairs. The number of CNVRs with a higher *V*_*ST*_ than top 1% pairs of populations as follows: 1033 in ASI-AFT pair, 31 in EAT-ASI pair, 21 in EAT-AFH pair, 15 in AFH-AFT pair and 2 in both ASI-AFH pair and EAT-AFT pair. The *V*_*ST*_ of pairs of 4 regional *B. taurus* populations: EAT, ASI, AFT and AFH were visualized as Manhattan plots (Fig. 3). Then, differences in rank of normalized copy number across 4 groups of *B. taurus* including ASI, EAT, AFT and AFH were tested using *Kruskal-Wallis* test. The population differentiation of CNVRs were determined by the following two criteria: *p*-value under 0.01 in *Kruskal-Wallis* test and pairwise *V*_*ST*_ in upper 1% which resulted in 910 CNVRs including 313 genes as candidates.

### Functional annotation of CNVR overlapping genes

Among 313 genes overlapped with 362 of population differentiated CNVRs, those with average copy number of four populations including EAT, AFT, AFH, and ASI were summarized in Table S3. The differentiated CNVRs were sorted in ascending order of chi-square from *Kruskal-Wallis* test. The average copy numbers for AFT, AFH, ASI, EAT groups were written under column for each group. Significantly under- or overrepresented PANTHER GO-slim molecular functions, GO-slim biological processes, or pathways were summarized in Table S4. Most of GO terms with significantly different representation between CNVRs and genome were overrepresented. Regulation associated terms including RNA polymerase II specific DNA binding, DNA-binding transcription factor, regulation of transcription by RNA polymerase II were overrepresented in differentiated CNVRs. Nervous system development and cell differentiation related terms were overrepresented, while immune response and structural constituent of ribosome were underrepresented. Among 72,840 of Autosomal QTLs, 7699 of QTLs overlapped with CNVRs. Five thousand two hundred fifty-two of QTLs overlapped with duplication CNVRs and 2642 of QTLs overlapped with deletion CNVRs. The representation of QTLs related to reproduction, milk and body weight were significantly different compared to total QTL. In reproduction related QTLs, the luteal activity was underrepresented while non-return rate, gestation length and calving ease were overrepresented on CNVRs. Most luteal activity QTL overlapping CNVRs overlapped with duplication while most gestation length QTL overlapped with deletion. The milk content related QTLs such as milk kappa-casein, glycosylated kappa-casein, unglycosylated kappa-casein percentage and milk potassium content were underrepresented on CNVRs. On the other hand, milk fat and yield QTLs were overrepresented. Body weight (yearling) and body weight gain QTLs were underrepresented on CNVRs.

## Discussion

Cattle have been spread with humans across the world after the domestication event in the Fertile Crescent in 10,000 YBP and Indus Valley in 8000 YBP. The genetic diversity of cattle population has been increased by its adaptation to various environments and demographic history including migration and introgression. For example, the population structure of African cattle has diversely changed from its earliest taurine-like population. Since the arrival of *B. indicus* around 700 AD [20, 21], the taurine × indicine cattle admixture event 750–1050 yr ago [11] and the introgression of African aurochs constructed the complex population structure of the current African cattle. Although population genetics of cattle has been studied extensively based on SNPs, the effects of CNVs on phenotypes and signatures of evolution were poorly understood.

CNVs cover a larger region of genome than SNPs and can impact gene function in multiple ways, including changing of gene structure and dosage, altering gene regulation and exposing recessive alleles [12]. Notably, genes overlapping CNVs were shown to have better correlations with differentially expressed genes than nearby SNPs, particularly when the CNV overlapped with exons [22]. Deletions in cattle genome can impact phenotype by interrupting genes and causing loss of biological function [23]. Duplicated genes in cattle genome were related to digestion, lactation, reproduction and immune system such as antigen processing and major histocompatibility genes [13, 24]. CNVs also have population genetic nature related to recombination, mutation, selection, and demography [25]. Generally, CNVs are more recent events than SNPs as they are still segregating within population, showing greater inter-individual variability [16]. These functional impacts and population genetic nature of CNVs have suggested that population differentiation of CNVs may contribute to the phenotypic variation between populations.

Recently, high quality cattle genome assemblies such as ARS-UCD1.2, UOA_Angus_1 and UOA_Brahman_1 increased reliability of CNV calling and resolution of breakpoint. Above all, Low et al. released haplotype-resolved genome assemblies of of *Bos taurus taurus* and *Bos taurus indicus*, and compared CNV between two subspecies [15]. They performed CNV calling using short reads from 38 animals of 7 cattle breeds.

We expanded samples to 336 individuals in 39 global cattle breeds in present study. We aligned short reads on ARS-UCD1.2 assembly to compare larger populations under unified criteria. We identified population stratification of autosome-wide CNVs based on NGS read mapping. Particularly, we included 206 individuals of 19 African cattle breeds in which their genome-wide CNV have been analyzed for the first time in this study.

The traditional classification for African indigenous cattle was based on phenotypes, especially the existence of cervico-thoracic hump. Based on this, some of the hybridized breeds were called Sanga (Zebu x Taurine) and Zenga (Zebu x Sanga). However, genome-wide SNP analysis has identified that the traditional classification did not reflect the genetic difference well [26, 27]. Our CNV based classification generally agreed with previous knowledge with exceptions in several individuals. There were two reasons for the disagreement. First, this study only covered copy number variation region, not the entire genome. Secondly, we compared the read mapping-based copy number, not the sequence itself. Nevertheless, overall concordance of clustering showed potential for population stratification using CNV.

In our CNV-based hierarchical clustering, most individuals were classified by their breeds, whereas some individuals including MAM01, MAM03, ANG09, ANK03 and part of Jersey individuals separated from their breeds. We inspected two possibilities to find the reason for discrepancy. First, we checked similarity between individuals in each breed. We referred to our previous study sharing large part of dataset [11]. The PCA plot and population structure from SNP genotype indirectly verified that there were no individuals that significantly distinguished from their breeds. Second, we checked the input vector of hierarchical clustering was the next suspicious factor after excluding the sample problem. It was too simple to represent CNV. The element of vector only considered existence of CNV on each CNVR, neglecting other properties such as length, breakpoint and copy number of CNV. However, when we tested two other vectors indicating type of CNV and normalized copy number of CNV, our original vector made a hierarchical tree which was the most concordant with breeds. We speculate that greater inter-individual variability of CNVs compared to SNPs and indels may have contributed to this discordance as well [16].

Mean *V*_*ST*_ and the number of CNVRs with high *V*_*ST*_ supported the ancestry of African cattle. AFT-EAT and AFH-ASI pairs were relatively similar while the AFT-ASI pair was mostly different. AFH exhibited high levels of shared CNV with ASI but not with AFT, probably because of the recency of their admixture which was around 150 generations ago [11]. Pairwise comparison of breed distinguished Muturu from other breeds, clustering them with the 3 Ethiopian zebu; Bagaria, Bale and Semien. The African taurine, especially Muturu, showed no evidence of admixture in previous studies assuming EAT and Asian-Australian indicine (AAI) as proxies for unadmixed taurine and indicine cattle, respectively [11]. Muturu was separated from EAT, ASI, and most of AFH except for Bale, Bagaria and Semien in pairwise mean *V*_*ST*_ clustering tree. Although the 3 Ethiopian breeds were clustered with Muturu, the mean pairwise *V*_*ST*_ did not imply their closeness to Muturu. The mean *V*_*ST*_ of Bale, Bagaria and Semien were 0.249, 0.244 and 0.251, respectively, which were all similar with the average 0.249. In addition, Italian taurine, Maremmana (0.132) and the Iberian indigenous taurine, Sayaguesa (0.189) and Pajuna (0.199) have lowest mean *V*_*ST*_ s against Muturu, which supported the shared ancestry between Muturu and Southern European taurine [11, 28].

Based on the *V*_*ST*_ and *Kruskal-Wallis* test on the copy number of CNVRs, 313 genes were identified as candidate genes under selection and adaptation. Of those, several genes were related to disease susceptibility and resistance. We identified significantly higher copy number of *HMGA2* in indicine than in taurine. The indicine-specific copy number gain of *HMGA2* was identified by chip-based methods and validated using qPCR in a previous study in which the *HMGA2* duplication in Nellore was suggested to be associated with navel length at yearling by haplotype-based GWAS (*p* = 1.01 × 10^−9^) [29]. Navel length at yearling is an economically important trait related to navel injuries in beef cattle. A pendulous navel increases risk of injuries and infection caused by friction against pasture [30]. During natural mating, bulls with long and pendulous navels are frequently exposed to injuries and trauma [31]. Expression of *HMGA2* gene is also responsible for body size by regulating myoblast proliferation and myogenesis. *HMGA2* directly regulates transcription of *IGF2BP2* (insulin like growth factor 2 mRNA binding protein 2), and *IGF2BP2* promotes myoblast growth. *IGF2BP2* regulates translation of *IGF1R* (insulin like growth factor 1 receptor), *c-Myc*, and/or *Sp1* by binding to their mRNA [32]. Among these genes related to muscle growth, *HMGA2*, *IGF2BP2* and *IGF1R* overlapped with our CNVRs. The copy numbers of CNVRs overlapping with *HMGA2* and *IGF1R* were significantly different between populations whereas those of *IGF2BP2* overlapping CNVRs were not. The copy number of *HMGA2* overlapping CNVR was gained in indicine population (EAT: 2.37, AFT: 2.48, AFH: 5.13, ASI: 8.85). On the contrary, the copy number of *IGF1R* overlapping CNVR was gained in taurine population and lost in indicine population (EAT: 3.28, AFT: 4.34, AFH: 0.92, ASI: 0.43). The knockout mice experiment suggested the positive impact of *HMGA2* expression on myoblast growth [32]. On the other hand, Chinese beef cattles with copy number loss of *IGF1R* had significantly better growth trait such as body weight, body height and hucklebone width [33]. In addition, *HMGA2* and *IGF1R* were strongly associated with size differences between dog breeds [34]. In conclusion, we suggest that differentiated copy number of *HMGA2* and *IGF1R* may be contributing to differences in body size between populations. Copy number variable genes overlapped with taurine-specific duplication such as *KRTAP9–1* and *KRTAP9–2*, and indicine-specific duplication such as *CATHL4* and *PRDM2* are related to pathogen- and parasite-resistance. The taurine-specific duplication of *KRTAP9–1* and *KRTAP9–2* corroborates the previous result of comparing copy number of them between European taurine and Asian zebu [18, 35]. They were also identified by aligning WGS short reads to three reference genome assemblies including ARS-UCD1.2, UOA_Angus_1 and UOA_Brahman_1 [15]. The keratin associated proteins were suggested to play a role in tick resistance [36, 37]. Since the cattle skin is the infestation site of tick, the structural protein keratin which makes up the outer layer of skin and hair could act as a barrier [38]. Also, the *PRDM2* gene was referred to play a role in resistance to disease and bacterial infection or cell-mediated immune response, especially paratuberculosis resistance in ruminants [39, 40]. The Paratuberculosis (Johne’s disease) caused by *Mycobacterium avium* subspecies *paratuberculosis* (*MAP*) brought about considerable economic losses worldwide. The GWAS cohort study about MAP infection in Holstein cattle identified strong signal of SNP and QTL adjacent to *PRDM2* gene [41]. Although the resistance to *MAP* has not yet been compared between taurine and indicine, the *PRDM2* gene overlapping indicine-specific duplication in our result can be the candidate region for further investigation on adaptation and selection related to paratuberculosis. The higher copy number of *CATHL4* in ASI than EUT was also identified in a previous study [18]. The bovine reference genome contains the expanded antimicrobial cathelicidin gene family whereas humans and mice have single copy [42]. Especially, the antimicrobial peptide, indolicidin encoded by *CATHL4* can induce autophagic cell death of *Leishmana donovani*, which is the causative parasite of Leishmaniasis [43]. .The antimicrobial ability which can influence Leishmaniasis lesion development of *CATH*-family genes was also proved by a knockout in mice [44]. Taken together, the population differentiated CNV on these genes may contribute to the increased parasite resistance in indicine compared to taurine.

ASI found across the tropical Indian subcontinent adapted to tropical environments characterized with heat stress as well as pervasive pathogen such as tick and parasite [45]. AFH whose ancestry of selection signature skewed toward indicine was also suggested to be adapted to heat stress by indicine introgression into local taurine [11]. In our analyses, one of the heat shock protein family coding gene, DNAJC18 is found to be overlapped with indicine-specific deletion, which is consistent with the CNVR identified in a previous study [46]. The DnaJ family binds to HSP70s for regulating their client capture and drives HSP70s toward specific client [47]. The significantly higher contribution of indicine ancestry [48] and selection signature in East African short horn zebu [49] imply that CNV on *DNAJC18* play a role in tropical adaptation and heat tolerance of zebu.

The olfactory function has evolved to alert animals of possible threats such as predators, and provides ability to avoid foods containing parasites, bacteria or chemicals [50]. It also assists animals in locating foods and potential mates [51]. .Olfactory receptors (ORs) play a key role in olfactory function, detecting odor molecules in the olfactory epithelium of the nasal cavity. The OR genes are the largest gene family in the mammalian genome, and there are 881 OR genes in cattle [52]. The OR genes are also characterized by extremely frequent gene duplications and losses [53]. In cattle, about 40% of OR loci are identified as CNVs. Therefore, the diversity and CNVs on OR genes in cattle could lead to breed specific differences in olfaction capacity [52]. In our result, several OR genes overlapped with the population differentiated CNVRs. There were *OR6C202*, *OR10AD1* and *OR5T2* on indicine-specific deletion, *OR8U3*, *OR4C1N*, *OR4C181*, *OR2AP1*, *OR9K2*, *OR4A16* and *OR5D14* on indicine-specific duplication, *OR4S1*, *OR5T2*, *OR8K1* and *OR5AS1* on ASI-specific deletion, *OR5M3* and *OR5AR1* on ASI-specific duplication and *OR8K3*, *OR5AS1* and *OR5L2* on African cattle specific duplication. As the significant variations in the number and repertoires of OR gene among vertebrates indicate that olfactory function has strongly influenced by natural selection our specific set of OR CNVs might give candidate CNVRs under selection.

Copy numbers of genes associated with quantitative traits related to productivity were frequently gained or lost on cattle genome. In our results, the Eukaryotic translation initiation factor 2 subunit 1 (*EIF2S1*) gene was overlapped with taurine-specific duplication from 7,927,275.2 to 79,278.2 kb in chromosome 10. Copy number on the CNVR in ASI-AFT pair was significant in *Kruskal-Wallis* test and their *V*_*ST*_ was 0.887. In previous study, *EIF2S1* was overlapped with CNVR specific to a high feed efficient group of Holstein [54], which suggests the contribution of the CNVR to different feed efficiency in beef cattle between *Bos taurus taurus* and *Bos taurus indicus* [55, 56]. The muscle development related gene *CTNNA1* was overlapped with indicine-specific deletion. This result was mostly agreed by Hu et al. [46] except for the lower copy number in our AFT individuals. The low copy number in *Bos taurus indicus* while normal or little change in *Bos taurus taurus* suggest that the sequence is likely to be specific to *Bos taurus taurus*. The *CTNNA1* gene has been described to be associated with myostatin expression level and transcription in skeletal muscle in Holstein-Friesian bulls [57]. Since myostatin plays an essential role in regulating skeletal muscle growth, the taurine-specific existence of *CTNNA1* gene would be one of the explanations for difference in meat productivity between *Bos taurus taurus* and *Bos taurus indicus*.

## Conclusions

In this study, we explored autosome-wide CNV of global cattle populations and estimated its differentiation between populations. Also, we improved accuracy and resolution of CNV detection compared to array-based methods and expanded our observation to African indigenous cattle of which CNV has not been investigated yet. The concordance between population differentiated CNVRs and previous association- and selection-studies supports the possible contributions of CNV to adaptation of cattle. However, our population-scale CNV analyses still have limited accuracy and resolution in detection due to high individual variability and using only one reference genome assembly. When using single reference genome, it would not represent some population enough and it is hard to distinguish CNVs whether minor or major. In further studies, we anticipate that the improvement of reference genome quality and additional high-quality genome assemblies can help solve these problems and enhance the evolutionary interpretation on genome-wide CNV of cattle.

## Methods

### Sample collection

The study population consisted of 336 individuals of 39 cattle breeds and 2 individuals of African Buffalo (*Syncerus caffer*, AFB). Most of individuals except for 10 Bale, 10 Bagaria, 10 Semien and 5 Afar were included in previous SNP-based study by Kim et al. [11]. Names of common individuals here followed the names used in the forementioned study [11]. Breeds of the two subspecies *Bos taurus taurus* and *Bos taurus indicus* were collected from Europe, Asia and Africa. Humpless taurine and crossbreeds such as Sanga (*Bos taurus taurus* x *Bos taurus indicus*) and Zenga cattle (Sanga x *Bos taurus indicus*) were collected from Africa. The 39 *Bos taurus* breeds were classified into four groups by their original region and subspecies as following: i) 102 individuals of European and Asian taurine (EAT) which included 10 Angus, 10 Holstein, 18 Hereford, 10 Jersey, 11 Simmental, 5 Eastern Finn, 5 Western Finn, 3 Maremmana, 2 Sayaguesa, 2 Pajuna, 1 Limia, 1 Maronesa, 1 Podolica and 23 Hanwoo; ii) 28 individuals of Asian indicine (ASI) which included 16 Brahman, 6 Nelore, 3 Gir, 1 Hariana, 1 Sahiwal and 1 Tharparkar; iii) 22 individuals of African tarurine (AFT) which included 9 Muturu and 13 N’Dama; and iv) 184 individuals of African humped cattle (AFH) which included African zebu and the crossbreeds sanga (zebu x taurine) and zenga (zebu x sanga). The African zebu consisted of 10 Arsi, 10 Bagaria, 10 Bale, 9 Barka, 20 Butana, 10 EthiopianBoran, 10 Goffa, 13 Kenana, 10 KenyaBoran, 10 Mursi, 9 Ogaden and 10 Semien. Sanga consisted of 14 Afar, 10 Ankole and 9 Sheko, and Zenga consisted of 9 Fogera and 11 Horro. Genomes of all individuals were sequenced by Illumina paired-end library and their additional information is described on Table S1. The publicly available sequences were downloaded from SRA with following project accession numbers; PRJNA574857 (Afar, African Buffalo, Arsi, Barka, Butana, Ethiopian Boran, Fogera, Goffa, Horro, Kenana, Mursi, N’Dama, Sheko), PRJNA318087 (Angus, Ankole, Jersey, Kenya, Boran, Kenana, N’Dama, and Ogaden), PRJNA514237 (Limia, Maremmana, Maronesa, Pajuna, Podolica, and Sayaguesa), PRJNA324822 (Brahman), PRJNA343262 (Brahman, Gir, Hereford, Nelore, and Simmental), PRJNA432125 (Brahman), PRJEB28185 (Eastern Finn, and Western Finn), PRJNA210523 (Hanwoo), PRJNA379859 (Hariana, Sahiwal, and Thaparkar), PRJNA210521 (Holstein), PRJNA386202 (Muturu), and PRJNA507259 (Nelore).

### Whole genome sequence alignment

After quality control checking of raw reads using FastQC-0.11.8 [58], adapter and low quality bases of reads were trimmed by Trimmomatic-0.39 [59]. After checking results of trimming and quality of trimmed reads, the trimmed reads were mapped using BWA-0.7.17 MEM [60] to reference genome ARS-UCD1.2 with Btau5.0.1 Y chromosome assembly. The output of sequence alignment map (SAM) were sorted, indexed and compressed to binary format (BAM) by Samtools-1.9 [24]. The duplicates in BAM files were marked using Picard 2.20.2 MarkDuplicates (https://broadinstitute.github.io/picard/) and the marked BAM files were used as input of variant calling. The alignment rate, coverage and mean depth were calculated using Sambamba [61].

### CNV calling and CNVR definition

CNVs of all samples were called with a bin size of 200 bp by CNVnator [62] and filtered with size (> 1 kb), *p*-value calculated using t-test statistics (< 0.001) and fraction of reads with zero mapping quality (MQ0 < 0.5). The CNVs in unplaced scaffolds were removed. A 50% reciprocal overlap between filtered CNVs was defined as copy number variation region (CNVR) using ‘CNV_overlap.py’ script on GitHub (https://github.com/bjtrost/TCAG-WGS-CNV-workflow) [63]. CNVRs found in more than two individuals were used for downstream analysis to minimize false-positive [64]. Copy number of each CNVR was calculated based on aligned read depth and normalized using CNVnator. The normalized copy number of neutral region from diploid autosome was assumed to be 2.0.

### Hierarchical clustering based on CNVR

To cluster individuals according to their CNV similarities, we made a vector of “0”s and “1”s for each individuals based on absence or presence of a specific CNVR in that particular individual. Hierarchical clustering with 1000 bootstrap resampling was performed on these vectors for every autosomal CNVR using pvclust with default option in R [65]. The ‘correlation’ and ‘average’ were used as distance measure and the agglomerative method, respectively. The approximately unbiased (AU) *p*-value was calculated by multiscale bootstrap resampling. The bootstrap probability (BP) *p*-value was calculated by ordinary bootstrap resampling based on unweighted pair-group average method (UPGMA).

### Population differentiation based on CNVR

The normalized copy number on CNVRs of all individuals was calculated using CNVnator [62]. *V*_*ST*_ of normalized copy number between a pair of breeds, was calculated as *V*_*ST*_ = (*V*_*T*_ − *V*_*S*_)/*V*_*T*_ where *V*_*T*_ is the total variance of normalized copy number among all individuals from both breeds and Vs is the average of variance within each breed, weighted by the number of individuals in the breed [66]. After excluding the 6 breeds with single individual, *V*_*ST*_ between pairs of 33 *Bos taurus* breeds and a buffalo breed were calculated. Mean *V*_*ST*_ of all autosomal CNVRs in each pair of breeds were visualized using pheatmap in R [67]. In addition, the *V*_*ST*_ of autosomal CNVRs were calculated between EAT, ASI, AFH and AFT. These results were visualized as Manhattan plots using qqman package in R [68]. After ranking the normalized copy numbers of all *B. taurus* individuals, *Kruskal-Wallist test* implemented in ‘kruskal.test’ R function were performed on all autosomal CNVRs to compare populations inlcuding EAT, ASI, AFH and AFT . Population differentiated CNVRs were defined as autosomal CNVRs with top 1% pairwise as well as *Kruskal-Wallis test p*-value less than 0.01.

### Functional annotation of genes overlapped with candidate CNVRs

Genes overlapped with autosomal CNVRs were annotated based on the reference genome ARS-UCD1.2 from NCBI RefSeq database [69]. In case of genes overlapped with multiple CNVRs, the CNVR with the most significantly different in *Kruskal-Wallis* test was written. Hypothetical, putative, predicted or uncharacterized genes and pseudo-genes were excluded. The information of functional annotation, gene ontology and pathway of the genes within the population differentiated CNVRs were identified using PANTHER classification system [70]. Comparing the list of genes overlapped with CNVRs with the all genes of *Bos taurus* in PATHER database [71], we tested the hypothesis whether the PANTHER GO-slim molecular function, GO-slim biological process, and pathway terms were under- or overrepresented in CNVRs using binomial test with *Bonferroni* corrections [70, 72]. The quantitative trait loci (QTL) underlying CNVRs were also identified using Cattle QTLdb of the reference genome ARS-UCD1.2 [73]. Under- or overrepresentation of autosomal QTL in autosomal CNVRs was tested using binomial test with *Bonferroni* corrections.

## Supplementary Information

**Additional file 1: Figure S1.** The number of population stratified CNVRs. *Venn* diagram of the number of population stratified CNVRs.

**Additional file 2: Table S1.** Sample information and alignment statistics.

**Additional file 3: Table S2.** Autosomal CNVRs sorted by their genomic region.

**Additional file 4: Table S3.** Genes overlapped with population differentiated CNVRs. Genes overlapped with significantly different CNVRs based on *Kruskal-Wallis* test result with <0.01 significance level and upper 1% *V*_*ST*_. Genes on CNVRs were sorted in ascending order by *p-*values. The pairs of populations with top 1% or top 0.1% *V*_*ST*_ and the average of copy number of CNVRs in populations including EAT, AFT, AFH and ASI are also indicated.

**Additional file 5: Table S4.** Over- / underrepresentation of PANTHER GO-slim molecular function, GO-slim biological process and pathway terms on CNVRs.

**Additional file 6: Table S5.** Over- / underrepresentation of QTLs on CNVRs.

**Additional file 7: Table S6.** Olfactory receptor genes overlapping population differentiated CNVRs.

## Data Availability

The newly generated sequences for 10 Bale, 10 Bagaria, 10 Semien and 5 Afar individuals are available from Sequence read archive (SRA) with the Bioproject accession number PRJNA698721. (https://www.ncbi.nlm.nih.gov/sra?linkname=bioproject_sra_all&from_uid=698721).

## Abbreviations

CNV: Copy number variation
SNP: Single nucleotide polymorphism
CNVR: Copy number variation region
EAT: Eurasian taurine
ASI: Asian indicus
AFH: African humped cattle
AFT: African taurine
