# Correction to: Population differentiated copy number variation of *Bos taurus, Bos indicus* and their African hybrids

**DOI:** 10.1186/s12864-022-08409-8

**Published:** 2022-03-15

**Authors:** Jisung Jang, Endashaw Terefe, Kwondo Kim, Young Ho Lee, Gurja Belay, Abdulfatai Tijjani, Jian-Lin Han, Olivier Hanotte, Heebal Kim

**Affiliations:** 1 Interdisciplinary Program in Bioinformatics, Seoul National University, Seoul, Republic of Korea; 2 Addis Ababa University, MCMB Department, Addis Ababa, Ethiopia; 3 International Livestock Research Institute (ILRI), Addis Ababa, Ethiopia; 4 Arsi University, Asella, Ethiopia; 5 Department of Agricultural Biotechnology and Research Institute of Agriculture and Life Sciences, Seoul National University, Seoul, Republic of Korea; 6 The Centre for Tropical Livestock Genetics and Health (CTLGH), The Roslin Institute, The University of Edinburgh, Edinburgh, UK; 7 CAAS-ILRI Joint Laboratory on Livestock and Forage Genetic Resources, Institute of Animal Science, Chinese Academy of Agricultural Sciences (CAAS), Beijing, China; 8 Livestock Genetics Program, International Livestock Research Institute (ILRI), Nairobi, Kenya; 9 School of Life Sciences, University of Nottingham, Nottingham, UK; 10 eGnome, Inc., Seoul, South Korea

**Correction to: BMC Genomics 22, 531 (2021)**

**https://doi.org/10.1186/s12864-021-07808-7**

Following publication of the original article [1], the authors reported that the following missing authors from the authorship list:

- Endashaw Terefe
- Gurja Belay
- Abdulfatai Tijjani
- Jian-Lin Han
- Olivier Hanotte

The corrected authorship list and the updated ‘Authors’ contributions’ and ‘Acknowledgements’ declarations are provided in this Correction article. The original article [1] has been updated.
