# Supplementary figures and images for: Population differentiated copy number variation of Bos taurus, Bos indicus and their African hybrids
Source: BMC Genomics. 2021 Jul 12;22:531. doi: 10.1186/s12864-021-07808-7 (PMC8276479; doi:10.1186/s12864-021-07808-7)

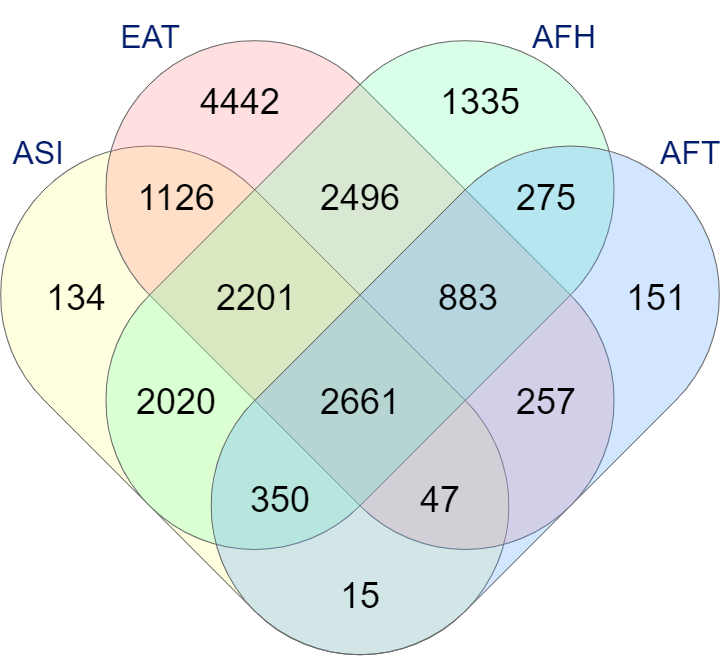

Supplement: Supplementary file 1 — Additional file 1: Figure S1. The number of population stratified CNVRs. Venn diagram of the number of population stratified CNVRs. [file 12864_2021_7808_MOESM1_ESM.png]
